# 3D joint T_1_/T_1__ρ_/T_2_ mapping and water‐fat imaging for contrast‐agent free myocardial tissue characterization at 1.5T

**DOI:** 10.1002/mrm.30397

**Published:** 2025-02-21

**Authors:** Michael G. Crabb, Karl P. Kunze, Simon J. Littlewood, Donovan Tripp, Anastasia Fotaki, Claudia Prieto, René M. Botnar

**Affiliations:** ^1^ School of Biomedical Engineering and Imaging Sciences King's College London London UK; ^2^ MR Research Collaborations Siemens Healthcare Limited Camberley UK; ^3^ School of Engineering Pontificia Universidad Católica de Chile Santiago Chile; ^4^ Millenium Institute for Intelligent Healthcare Engineering Santiago Chile; ^5^ Institute for Biological and Medical Engineering Pontificia Universidad Católica de Chile Santiago Chile; ^6^ Institute for Advanced Study Technical University of Munich Garching Germany

**Keywords:** $T_{1\rho }$ mapping, $T_{1}$ mapping, $T_{2}$ mapping, 3D multi‐parametric MRI, cardiac MRI, myocardial tissue characterisation

## Abstract

**Purpose:**

To develop a novel, free‐breathing, 3D joint $T_{1}$/$T_{1\rho }$/$T_{2}$ mapping sequence with Dixon encoding to provide co‐registered 3D $T_{1}$, $T_{1\rho }$, and $T_{2}$ maps and water‐fat volumes with isotropic spatial resolution in a single ≈7 min scan for comprehensive contrast‐agent‐free myocardial tissue characterization and simultaneous evaluation of the whole‐heart anatomy.

**Methods:**

An interleaving sequence over 5 heartbeats is proposed to provide $T_{1}$, $T_{1\rho }$, and $T_{2}$ encoding, with 3D data acquired with Dixon gradient‐echo readout and 2D image navigators to enable 100% respiratory scan efficiency. Images were reconstructed with a non‐rigid motion‐corrected, low‐rank patch‐based reconstruction, and maps were generated through dictionary matching. The proposed sequence was compared against conventional 2D techniques in phantoms, 10 healthy subjects, and 1 patient.

**Results:**

The proposed 3D $T_{1}$, $T_{1\rho }$, and $T_{2}$ measurements showed excellent correlation with 2D reference measurements in phantoms. For healthy subjects, the mapping values of septal myocardial tissue were $T_{1}=1060\pm 48\;\text{ms}$, $T_{1\rho }=48.1\pm 3.9\;\text{ms}$, and $T_{2}=44.2\pm 3.2\;\text{ms}$ for the proposed sequence, against $T_{1}=959\pm 15\;\text{ms}$, $T_{1\rho }=56.4\pm 1.9\;\text{ms}$, and $T_{2}=47.3\pm 1.5\;\text{ms}$ for 2D MOLLI, 2D $T_{1\rho }$‐prep bSSFP and 2D $T_{2}$‐prep bSSFP, respectively. Promising results were obtained when comparing the proposed mapping to 2D references in 1 patient with active myocarditis.

**Conclusion:**

The proposed approach enables simultaneous 3D whole‐heart joint $T_{1}$/$T_{1\rho }$/$T_{2}$ mapping and water/fat imaging in ≈ 7 min scan time, demonstrating good agreement with conventional mapping techniques in phantoms and healthy subjects and promising results in 1 patient with suspected cardiovascular disease.

## 1 INTRODUCTION

The use of gadolinium‐based contrast agents is clinically accepted for the detection of myocardial scar.^1^ However, in patients with renal dysfunction or contrast agent allergy, a non‐contrast‐enhanced approach would be of great value. Native $T_{1}$, $T_{1\rho }$, and $T_{2}$ myocardial mapping have shown promising results in noninvasive detection of a range of different cardiomyopathies. This includes assessment of myocardial infarction and focal and diffuse fibrosis for $T_{1}$,^2^, ^3^, ^4^, ^5^, ^6^ chronic myocardial infarction and fibrosis for $T_{1\rho }$,^7^, ^8^, ^9^, ^10^ and assessment of inflammation and edema using $T_{2}$,^11^, ^12^ thus helping to distinguish between acute and chronic infarction and providing complementary information for diagnosis. Furthermore, characterization of fibrofatty infiltration of the myocardium has been shown to be clinically relevant.^13^, ^14^

Myocardial maps are commonly acquired clinically through single‐parameter 2D mapping sequences, including MOLLI^15^ and SASHA^16^ for $T_{1}$, and $T_{2}$‐prep bSSFP^17^ for $T_{2}$. These are usually acquired under a breath‐hold per short‐axis slice, yielding limited heart coverage and low spatial resolution. Also, additional heartbeats (HBs) are required to ensure magnetization recovery between preparation pulses. $T_{1\rho }$ mapping is less common clinically, but 2D breath‐hold sequences have recently been proposed.^18^, ^19^ However, these suffer from the same problems as previously mentioned for 2D mapping.

3D free‐breathing, single‐parameter mapping techniques have been proposed to remove the requirement of breath‐holds and increase heart coverage and spatial resolution. These include respiratory‐gated 3D mapping,^20^, ^21^ and respiratory motion‐compensated 3D mapping^22^, ^23^, ^24^ using 2D image navigators (iNAVs) and translational respiratory motion correction, leading to 100% respiratory scan efficiency and predictable scan time. However, these do not account for non‐rigid deformation of the heart through the respiratory cycle, but non‐rigid motion‐compensated reconstruction can be achieved through iNAV‐based translation motion estimation in concert with respiratory binning.^25^, ^26^, ^27^

Multi‐parametric mapping sequences, where several parameters are quantified in a single scan, may improve accuracy and protocol efficiency, simplify segmentation and analysis, and avoid misregistration over sequentially acquired sequences.^28^ For 2D multi‐parametric mapping, the most common parameters mapped are $T_{1}$ and $T_{2}$,^29^, ^30^, ^31^ but $T_{1\rho }$ has more recently been proposed in 2D joint $T_{1}$/$T_{1\rho }$ mapping at 3T,^32^ 2D $T_{1}$/$T_{2}$/$T_{1\rho }$ Magnetic Resonance Fingerprinting (MRF) at 1.5T,^33^ and 2D multi‐parametric $T_{1}$/$T_{2}$/$T_{1\rho }$ mapping at 3T.^34^ Fat quantification has been additionally incorporated in 2D $T_{1}$/$T_{2}$ MRF.^35^, ^36^ For 3D joint mapping, 3D joint $T_{1}$/$T_{2}$ mapping sequences that acquire data in the steady state have been proposed,^37^, ^38^ and a 3D joint $T_{1}$/$T_{2}$ mapping technique has recently been proposed that aims to provide co‐registered 3D $T_{1}$ and $T_{2}$ maps and water‐fat volumes with isotropic spatial resolution at 1.5T.^28^ A 3D joint $T_{1}$/$T_{1\rho }$ mapping technique has also been proposed at 3T.^39^

The aim of this work was to develop a novel, free‐breathing, 3D whole‐heart joint $T_{1}$/$T_{1\rho }$/$T_{2}$ mapping sequence with Dixon encoding to provide co‐registered 3D $T_{1}$, $T_{1\rho }$, and $T_{2}$ maps and water‐fat volumes with isotropic spatial resolution in a single ≈7 min scan for comprehensive contrast‐agent‐free myocardial tissue characterization and simultaneous evaluation of the cardiac and coronary anatomy. A complementary co‐registered $T_{2}$‐prepared bright‐blood water volume is obtained for whole‐heart anatomy visualization.

## 2 METHODS

### 2.1 Pulse sequence

**FIGURE 1:**
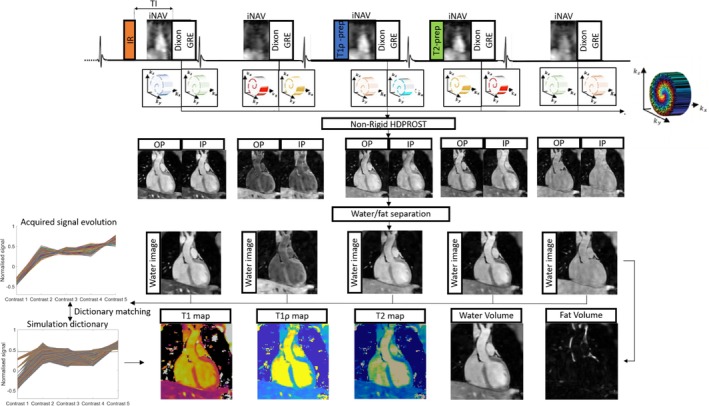
3D joint $T_{1}$/$T_{1\rho }$/$T_{2}$ mapping and water/fat imaging sequence schematic diagram. Volume 1: IR (TI=250ms), Volume 2: no‐preparation, Volume 3: $T_{1\rho }$‐preparation (spin‐lock duration =40ms and spin‐lock amplitude =400Hz), Volume 4: $T_{2}$‐preparation (duration =40ms), Volume 5: no preparation. Non‐rigid motion correction with patch‐based regularization is used to reconstruct 3D IP/OP HB images; water and fat images are generated from IP/OP images through a fat‐water separation algorithm, and $T_{1}$/$T_{1\rho }$/$T_{2}$ maps are estimated from water images by dictionary matching via a Bloch simulation of the proposed 3D sequence.

The proposed ECG‐triggered 3D joint $T_{1}$/$T_{1\rho }$/$T_{2}$ research sequence (Figure 1) consisted of a repeating set of preparation modules defined over 5 HBs: a 180° adiabatic IR pulse, no preparation, $T_{1\rho }$ preparation, $T_{2}$ preparation, and no preparation. For $T_{1\rho }$ preparation, four spin‐locking pulses with alternating phases and two rectangular refocusing pulses with opposite phases were deployed to make $T_{1\rho }$ preparation more robust to $B_{0}$ and $B_{1}$ inhomogeneities.^40^, ^41^ A spin‐lock duration (TSL) =40ms and spin‐lock amplitude (SLA) =400Hz were used. For $T_{2}$ preparation, two adiabatic refocusing pulses were deployed to make preparation more robust to $B_{0}$ and $B_{1}$ inhomogeneities^42^ with duration =40ms. The 5th idle HB is to compensate for $T_{1}$ recovery for the next shot of the IR preparation and provide greater $T_{1}$ sensitivity for longer $T_{1}$ or heart‐rates (HRs). Five interleaved volumes were acquired with a 2‐point bipolar Dixon RF‐spoiled gradient recalled echo (GRE) readout with a variable‐density 3D Cartesian trajectory with spiral profile order (VD‐CASPR).^43^ A golden angle step within and across contrasts was used to ensure incoherent undersampling artifacts both within and across contrasts, with an acceleration factor = 6 per contrast chosen. 2D iNAVs^44^ were acquired prior to each 3D acquisition to enable beat‐to‐beat respiratory motion estimation and correction.

#### 2.1.1 Dictionary generation and simulations

Numerical simulations of the proposed sequence were performed to generate signal dictionaries, as well as to test the accuracy and precision of the sequence (Supporting Information, Figure S1). A 1D Bloch equation simulation of the longitudinal magnetization, assuming perfect spoiling, was implemented in MATLAB (The MathWorks Inc., Natick, MA, USA) to generate a signal at each TR. The simulation consisted of 3 sets of 5 HBs, with the first 2 sets simulated to reach the steady‐state magnetization. The signal from the last set of 5 HBs was averaged over the first 30% of imaging readout (since data was acquired with centric k‐space reordering) to yield a dictionary with 5 points. Each signal evolution in the dictionary (corresponding to a unique parameter combination) was normalized. For *in‐vivo* data, subject‐specific dictionaries were generated using the mean RR interval and acquisition window. The dictionary used the following parameter combinations ([start:step:end]): $T_{1}=[50:50:600,600:15:1800,1800:50:2200,2200:100:3000]\text{ms}$, $T_{1\rho }=[5:5:20,20:1.5:80,80:4:100,100:10:300,300:100:600]\;\text{ms}$, $T_{2}=[5:5:20,20:1.5:80,80:4:100,100:10:300,300:100:600]\text{ms}$, constrained such that $T_{2}\leq T_{1\rho }$ and $T_{1\rho }\leq T_{1}$.

### 2.2 Image reconstruction

#### 2.2.1 Motion correction

2D iNAVs were used to generate a low‐resolution 2D image in coronal orientation at each HB. Beat‐to‐beat translational Right‐Left (RL)/Foot‐Head (FH) respiratory motion fields were estimated from the iNAVs via normalized cross‐correlation. The estimated FH translational motion field was used to assign the acquired k‐space data for each contrast into a set of B=4 respiratory bins, and intra‐bin 2D translational motion (RL and FH) correction was performed by correcting the k‐space data for each bin to the same respiratory position (taken here as the bin centre).^25^ For non‐rigid motion estimation, binned images were reconstructed with iterative SENSE with soft‐binning^25^ using the respiratory binned k‐space data from $4^{\text{th}}$ contrast, $1^{\text{st}}$ echo. Non‐rigid registration using free‐form deformation^45^ was deployed on binned images, using end expiration bin as reference,^46^ yielding motion fields ${\left\{U_{b}\right\}}_{b=1}^{B}$. These were used to build the following motion‐correction encoding operator, $E^{\left(c\right)}$,

(1) $$E^{\left(c\right)}=\overset{B}{\underset{b=1}{\sum }}A_{b}^{\left(c\right)}ℱSU_{b},$$

where $A_{b}^{\left(c\right)}$ is the binned sampling mask for bin b=1,…,B and contrast c=1,…,C, ℱ is the Fourier Transform, and S the coil sensitivities.^47^ Here, C=10 corresponds to the out‐of‐phase (OP) and in‐phase (IP) echoes for all 5 interleaved contrast acquisitions.

#### 2.2.2 Patch‐based, low‐rank reconstruction

The 10 3D image contrasts were reconstructed using non‐rigid motion correction with patch‐based multi‐dimensional low‐rank regularization (HD‐PROST).^27^, ^48^ Image reconstruction was posed as minimisation of the following Lagrangian

(2) $$\begin{array}{ll} ℒ(X,T,Y) & =||EX-K|{|}_{F}^{2}+\lambda \underset{v}{\sum }||T_{v}|{|}_{*}\\ & \;+\frac{\mu }{2}\underset{v}{\sum }||T_{v}-P_{v}(X)-\frac{1}{\mu }Y|{|}_{F}^{2},\; \end{array}$$

where X are the contrast images, E = $\left(E^{\left(1\right)},\ldots ,E^{\left(c\right)}\right)$ is the multi‐contrast encoding operator, K is the multi‐coil multi‐contrast translational motion‐corrected k‐space data, and $P_{v}(X)$ is the patch selection operator for voxel v, $T_{v}$ is a tensor of patches similar to the patch at the vth voxel, Y is a Lagrange multiplier, μ, λ are regularization parameters, and $||\cdot |{|}_{*/F}$ are nuclear/Frobenius norms. The Alternating Direction Method of Multipliers (ADMM) method^49^ was used to solve this problem. For each outer iteration of ADMM, the minimization is performed by the sequential solution of the following 3 sub‐problems: (i) regularized MR reconstruction (minimization of (2) wrt X, with T,Y fixed) solved using iterative SENSE (5 iterations); (ii) denoising (minimization of (2) wrt T, with X,Y fixed) solved using higher‐order singular value decomposition and truncation, and (iii) Lagrange multiplier update of T. These sub‐problems are repeated over 5 outer ADMM iterations and regularization parameters, λ=0.04 and μ=0.1 are selected. For the patch‐based denoising step 2, other parameters selected include a voxel search window =20×20×20, a voxel patch size =5×5×5, a number of selected similar patches =20, and a voxel patch offset = 4 when searching for similar patches (see^27^, ^50^ for definitions and further details).

#### 2.2.3 Water/fat separation

Water and fat images for all 5 interleaved contrast acquisitions were subsequently estimated from the corresponding reconstructed IP/OP images via background phase correction to eliminate errors due to $B_{0}$ inhomogeneity using the B0‐NICE method.^51^

#### 2.2.4 Mapping

For the generation of signal dictionaries, a simulation of the sequence was implemented in MATLAB as outlined in section 2.1.1. The signal polarity was estimated through background phase‐removal using the $4^{\text{th}}$ contrast IP echo image,^52^ and the polarity‐restored water images were normalized across contrasts for each image voxel. Matching was performed voxel‐wise by selecting the parameter corresponding to the maximum inner‐product of the normalised measured signal with the dictionary.

### 2.3 Experiments

The proposed 3D joint $T_{1}$/$T_{1\rho }$/$T_{2}$ sequence was tested in phantoms, healthy subjects, and patients. Data was acquired on a 1.5T MR scanner (MAGNETOM Aera, Siemens Healthineers AG, Erlangen, Germany) with an 18‐channel chest array and a 32‐channel spine array. Written informed consent was obtained from all participants before undergoing the MR scan, and the study was approved by the institutional review board.

#### 2.3.1 Phantoms

Data was acquired in a $T_{1}$‐MES phantom^53^ and an in‐house phantom with vials of variable agar/${\text{NiCl}}_{2}$ concentration.^33^ The mapping values obtained with the proposed approach were compared with 2D IR‐prep Spin‐Echo (IR SE), 2D $T_{1\rho }$‐prep SE, and 2D $T_{2}$ SE measurements for both accuracy and precision.

The proposed 3D joint $T_{1}/T_{1\rho }$/$T_{2}$ mapping was acquired with 2‐point bipolar Dixon GRE read‐out (${\text{TE}}_{1}/{\text{TE}}_{2}/\text{TR}=2.38/4.76/6.71\text{ms}$, flip angle (FA) =8°, bandwidth (BW) =453Hz/pixel), iNAV (14(×2)‐echoes, FA=3°), TI=250ms, VD‐CASPR acceleration =×6/contrast with centric k‐space reordering, $\text{FOV}=320\times 320\times 20{\text{mm}}^{3}$, resolution =2mm isotropic. 16 segments were acquired per HB, corresponding to an acquisition window =107ms.

For 2D IR SE, imaging parameters included TE/TR=11/10000ms, FA=90°, resolution $=2\times 2{\;\text{mm}}^{2}$, slice‐thickness =8mm, BW=130Hz/pixel. 9 IR‐preps with TIs=[50,350,650,950,1250,1550,1850,2150,2450]ms were used. A 2‐parameter fit, $\left(T_{1},M_{0}\right)$, to the following signal equation $S(T_{1},M_{0},\text{TI},\text{TR})=M_{0}(1-2\exp(-\text{TI}/T_{1})+\exp(-\text{TR}/T_{1}))$ was used, solved using the Levenberg‐Marquardt algorithm.

For 2D $T_{1\rho }$‐prep SE, imaging parameters included TE/TR=12/5000ms, FA=90°, resolution $=2\times 2{\text{mm}}^{2}$, slice‐thickness =8mm, BW=130Hz/pixel. 8 $T_{1\rho }$‐preps with TSL =[2,14,26,38,50,62,74,86]ms and SLA =400Hz were used. A 1‐parameter fit $T_{1\rho }$ to the log of the following signal equation, $S(T_{1\rho },M_{0},\text{TSL})=M_{0}\exp(-\text{TSL}/T_{1\rho })$, was obtained through linear least‐squares fitting.

For 2D $T_{2}$ SE, imaging parameters included TR=10000ms, FA=90°, resolution $=2\times 2{\text{mm}}^{2}$, slice‐thickness =8mm, BW=130Hz/pixel. 8 images with TEs=[12,27,42,57,72,87,102,117]ms were acquired. A 1‐parameter fit $T_{2}$ to the log of the following signal equation, $S(T_{2},M_{0},\text{TE})=M_{0}\exp(-\text{TE}/T_{2})$, was obtained through linear least‐squares fitting.

#### 2.3.2 Healthy subjects

Data was acquired in 10 healthy subjects. The accuracy and precision of values from the proposed mapping sequence were compared to 2D MOLLI,^15^ 2D $T_{1\rho }$‐prep bSSFP,^24^ and 2D $T_{2}$‐prep bSSFP^17^ mapping sequences at mid‐ventricular short‐axis (SAx) slice. Two‐tailed Student's t‐tests (p<0.05) were used to analyze differences in mean/SD $T_{1}$/$T_{1\rho }$/$T_{2}$ values.

For the proposed 3D joint $T_{1}/T_{1\rho }$/$T_{2}$ mapping, a subject‐specific slice selection covering the heart ($\text{FOV}=320\times 320\times 96-120{\text{mm}}^{3}$) was planned following acquisition of a cardiac localiser. Imaging was performed at mid‐diastole with a trigger delay and acquisition window informed through acquisition of a free‐breathing 4‐chamber CINE. The acquisition window was 124±16ms. The remaining imaging parameters matched those used for phantoms. The total acquisition time was 7.3±0.8 mins.

For 2D MOLLI, the MOLLI 5(3)3 sequence^15^ was deployed with bSSFP readout with TE/TR=1.12/2.8ms, FA=35°, resolution $=1.6\times 1.6{\text{mm}}^{2}$, slice‐thickness =8mm, and BW=1085Hz/pixel. The $T_{1}$ weighted images and maps were obtained inline on the scanner.

For 2D $T_{1\rho }$, an ECG‐triggered 2D $T_{1\rho }$‐prepared bSSFP reference sequence^24^ was acquired during a breath‐hold at mid‐diastole and consists of the acquisition of 5 $T_{1\rho }$‐prepared images with TSL =[1,10,20,35,50]ms and SLA =400Hz. A saturation (SAT) pulse was deployed at each HB with a constant delay prior to $T_{1\rho }$‐preparation. A bSSFP readout was used with TE/TR=1.2/3.3ms (partial Fourier in readout direction), FA=50°, and parallel imaging using GRAPPA (acceleration factor ×2), $\text{FOV}=300\times 300{\text{mm}}^{2}$, resolution $=2\times 2{\text{mm}}^{2}$, slice‐thickness =8mm. A fixed 170ms acquisition window was used, corresponding to 3 HBs/contrast and a scan time of 15 HBs per short‐axis slice. Mono‐exponential fitting was used to generate $T_{1\rho }$ maps from the reconstructed $T_{1\rho }$‐weighted images, working through image voxel by voxel.

For 2D $T_{2}$, the 2D $T_{2}$‐prepared bSSFP sequence^17^ was deployed with 3 $T_{2}$‐prepared images acquired ($T_{2}$‐prep durations =[0,25,55]ms) and 3 recovery beats. A bSSFP readout with TE⁄TR=1.06⁄2.49ms, FA=70°, resolution $=1.9\times 1.9{\text{mm}}^{2}$, slice‐thickness =8mm, BW=1185Hz/pixel, and linear k‐space reordering was used. The $T_{2}$ weighted images and maps were obtained inline on the scanner.

#### 2.3.3 Patients

The proposed 3D joint $T_{1}/T_{1\rho }$/$T_{2}$, 2D MOLLI, 2D $T_{1\rho }$‐prep bSSFP, and 2D $T_{2}$‐prep bSSFP sequences were acquired in 1 patient with suspected cardiovascular disease. Acquisition parameters matched those used for healthy subjects. For the patient, $T_{2}$‐weighted Turbo Spin Echo ($T_{2}$w‐TSE)^54^ (1.5×1.5×8 ${\text{mm}}^{3}$) was additionally acquired in several SAx views.

## 3 RESULTS

### 3.1 Phantoms

**FIGURE 2:**
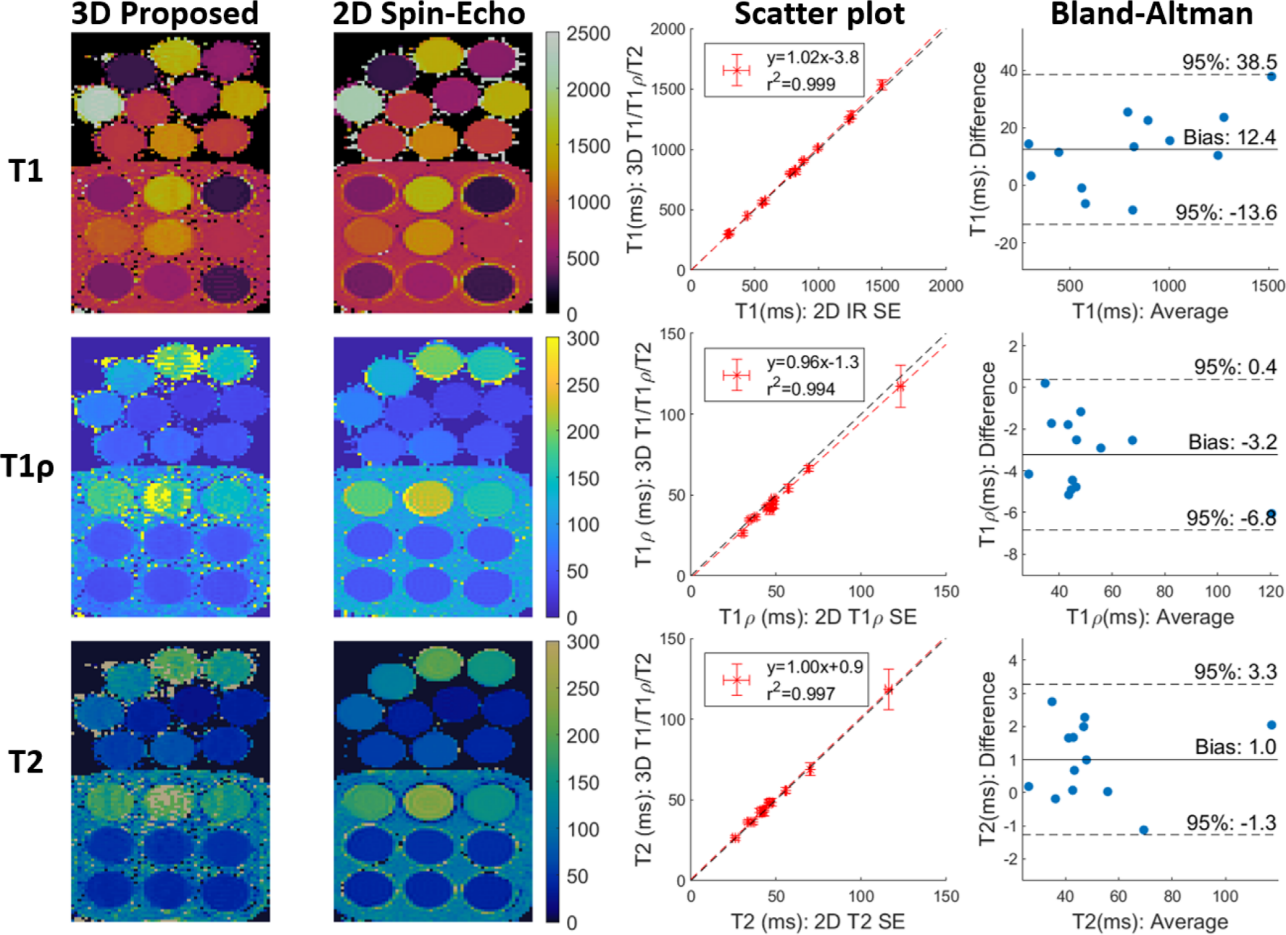
Left: Comparison of proposed 3D $T_{1}$/$T_{1\rho }$/$T_{2}$ mapping to 2D IR SE, 2D $T_{1\rho }$‐prep SE, and 2D $T_{2}$ SE (simulated heart‐rate (HR)=60 bpm). The top 10 vials are from the in‐house phantom, and the bottom 9 vials are from the $T_{1}$‐MES phantom. Middle: Scatter plot comparison of proposed 3D $T_{1}$/$T_{1\rho }$/$T_{2}$ to 2D SE mapping references. The black and red lines are identity lines and linear fits, respectively. Right: Bland‐Altman plot comparison of proposed 3D $T_{1}$/$T_{1\rho }$/$T_{2}$ to 2D SE mapping references.

For phantom results, we first show a comparison of the proposed 3D $T_{1}$/$T_{1\rho }$/$T_{2}$ mapping sequence and 2D IR SE, 2D $T_{1\rho }$‐prep SE, and 2D $T_{2}$ SE references with a simulated HR = 60 bpm in Figure 2. High linear correlations of y=1.02x−3.8 ($r^{2}=0.999)$, y=0.96x−1.3 ($r^{2}=0.994)$, and y=1.00x+0.9 ($r^{2}=0.997)$ were observed when comparing the 3D joint $T_{1}$/$T_{1\rho }$/$T_{2}$ sequence to 2D IR SE, 2D $T_{1\rho }$‐prep SE, and 2D $T_{2}$ SE, respectively. Small biases of +12ms, −3.2ms and +1.0ms for proposed $T_{1}$, $T_{1\rho }$, and $T_{2}$ were observed, respectively. Vials with $T_{1}\geq 1500\text{ms}$, $T_{1\rho }\geq 150\;\text{ms}$, and $T_{2}\geq 150\text{ms}$ were rejected prior to fitting to focus on the typical myocardial tissue value range. Generally, vials with large $T_{1}$, $T_{1\rho }$ or $T_{2}$ were less accurate and precise, since the sequence is designed for values within the myocardium. In particular, for $T_{1\rho }$ and $T_{2}$, the proposed sequence only uses a single $T_{1\rho }$‐prep and $T_{2}$‐prep, each with a 40 ms duration, and, for $T_{1}$, there are only 5 HBs between IR pulses. Experiments were also performed to compare the proposed mapping sequence with 2D SE references over a range of simulated HRs (Supporting Information, Figure S2).

### 3.2 Healthy subjects

**FIGURE 3:**
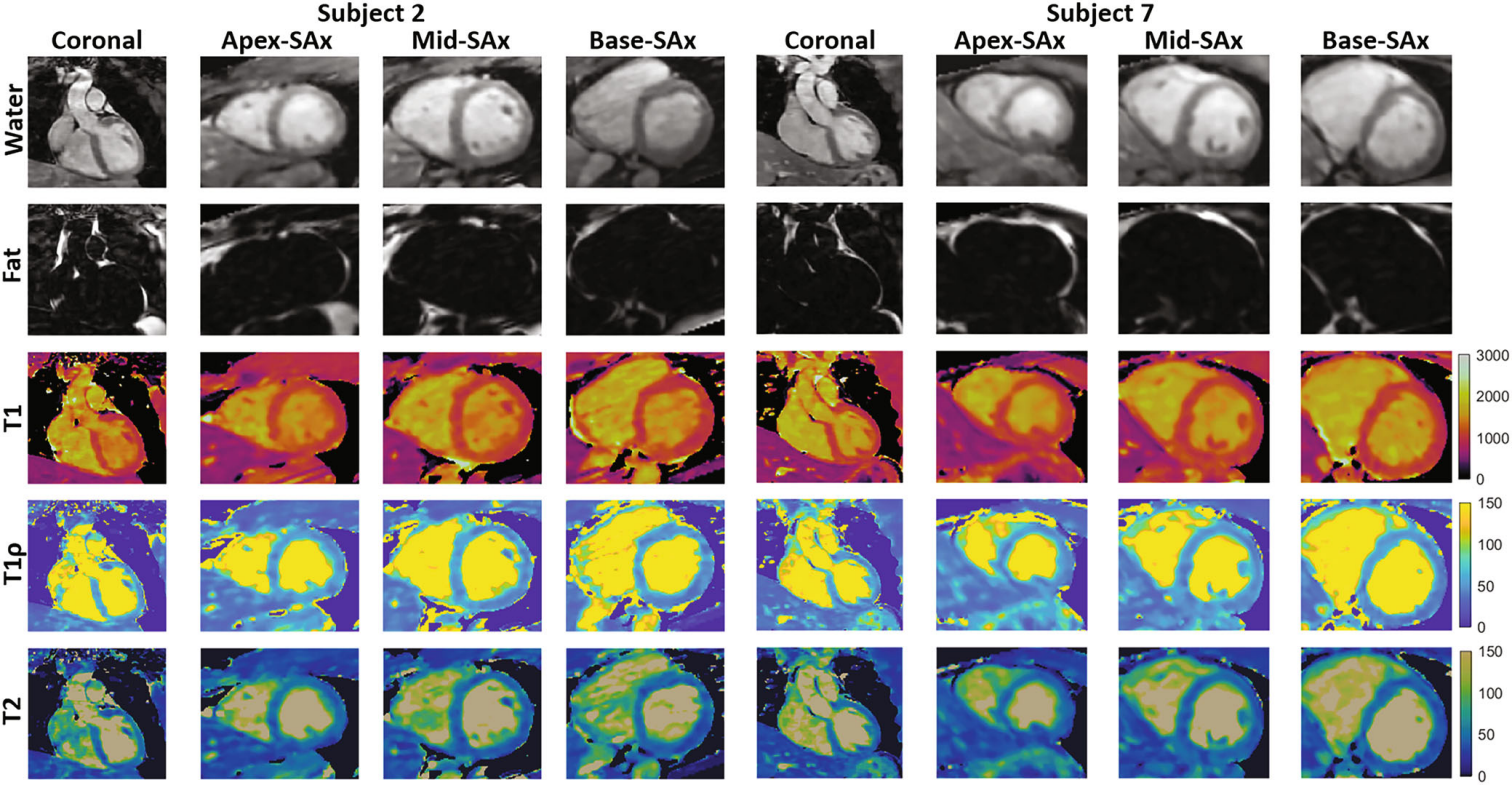
Water (4th HB), Fat (4th HB), $T_{1}$, $T_{1\rho }$, and $T_{2}$ mappings from the proposed 3D joint $T_{1}$/$T_{1\rho }$/$T_{2}$ mapping sequence with Dixon encoding showing coronal, apex‐, mid‐, and base‐ventricular short‐axis (SAx) views through the 3D reconstruction in 2 healthy subjects.

**FIGURE 4:**
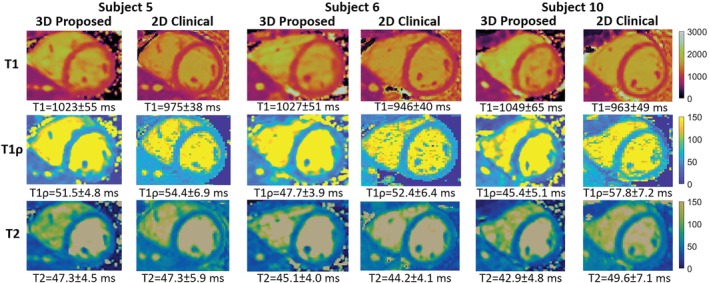
Comparison of 3D $T_{1}$/$T_{1\rho }$/$T_{2}$ to 2D references at mid‐ventricular short‐axis (SAx) for 3 representative healthy subjects. The mean and standard deviation of mid‐ventricular SAx septal myocardium values are displayed.

Figure 3 shows different views of the 3D volume of Water ($4^{\text{th}}$ contrast), Fat ($4^{\text{th}}$ contrast), $T_{1}$, $T_{1\rho }$, and $T_{2}$ maps in 2 representative healthy subjects. Good quality water and fat volumes and maps are observed, with clear definition of the myocardium in both subjects. Water and fat contrast images for each of the 5 contrast interleaved acquisitions for a representative subject are shown in Supporting Information, Figure S3. A 16‐segment AHA model^55^ was calculated on the reformatted SAx slices for each of the reconstructed 3D $T_{1}$, $T_{1\rho }$, and $T_{2}$ maps for all healthy subjects. Mean and Standard Deviation (SD) were calculated for each segment of the AHA model. Figure 4 illustrates mid‐ventricular SAx views of the proposed 3D $T_{1}$/$T_{1\rho }$/$T_{2}$ maps, 2D MOLLI, 2D $T_{1\rho }$‐prep bSSFP, and 2D $T_{2}$‐prep bSSFP for 3 representative healthy subjects, and good qualitative agreement is observed between the 3D proposed and 2D reference maps. SAx views of the 3D proposed maps extending from apex to base, high‐resolution Bull's eye plots, and histograms of measured left‐ventricle mapping values for a representative healthy subject are demonstrated for $T_{1}$, $T_{1\rho }$, and $T_{2}$ in Supporting Information Figure S4, S5, and S6 respectively.

**FIGURE 5:**
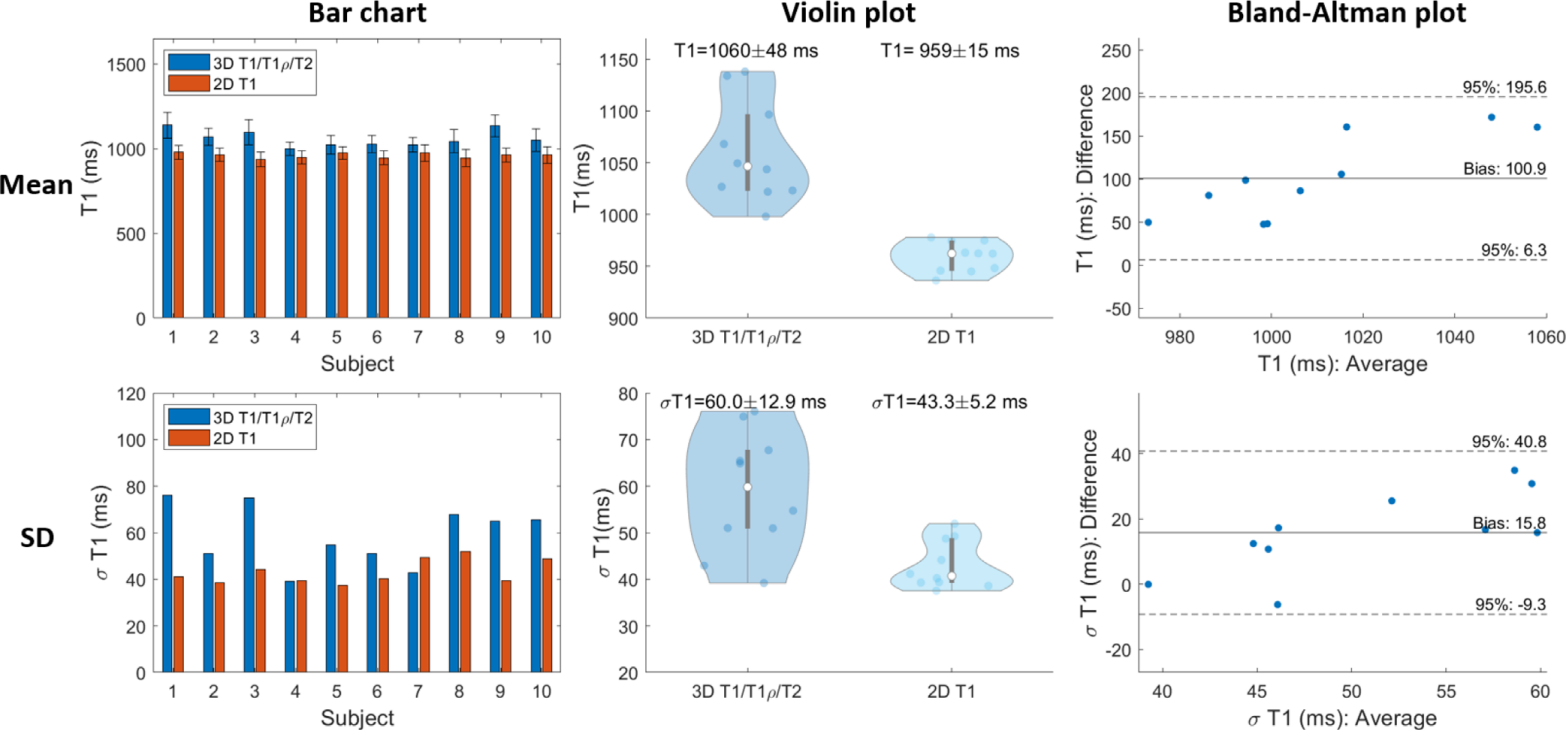
Comparison of mid‐ventricular short‐axis septal myocardium values for proposed $T_{1}$ and 2D MOLLI across healthy subjects. Top/Bottom figures are the Mean/Standard Deviation (SD) of myocardial values Left to Right are Bar chart, Scatter, Violin and Bland‐Altman plots.

**FIGURE 6:**
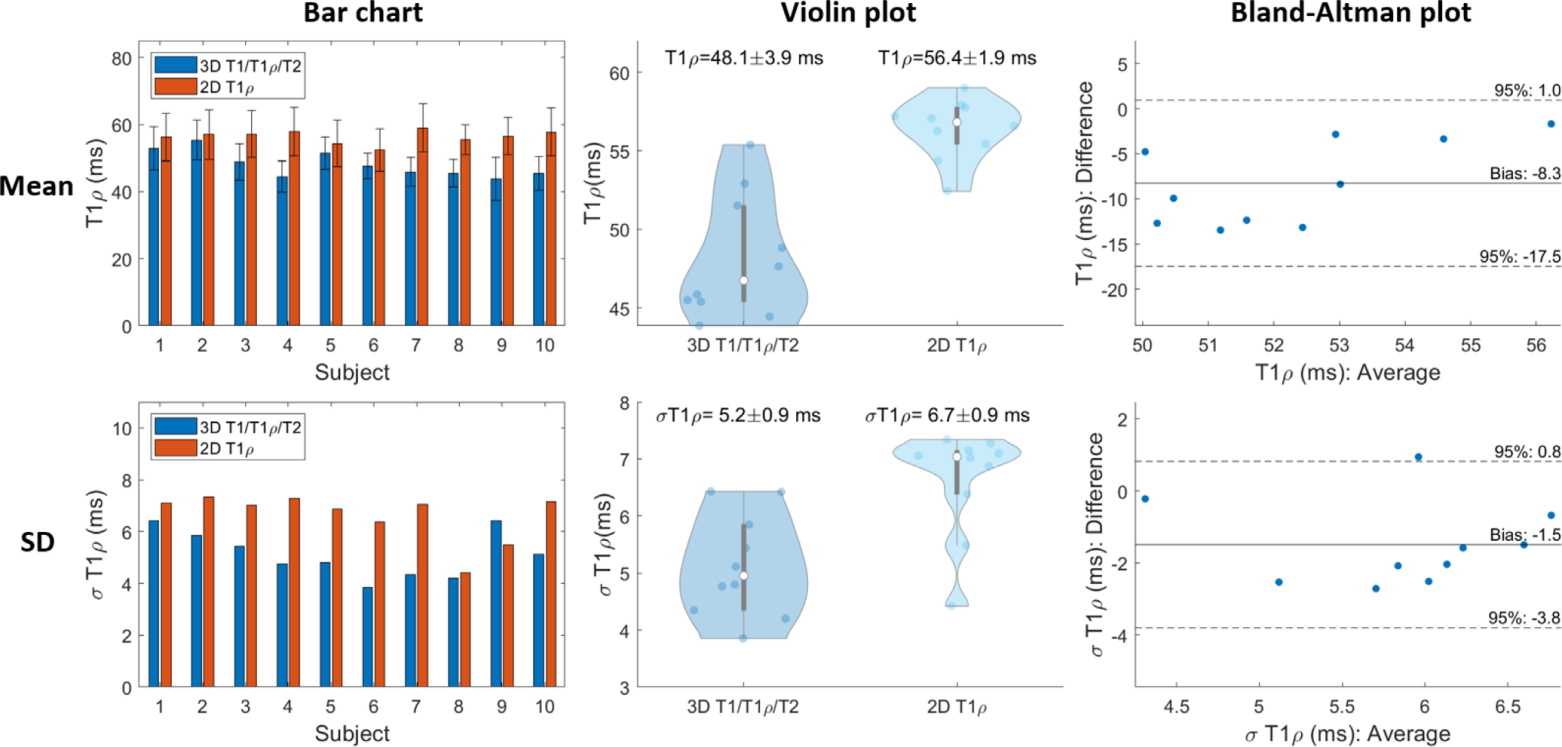
Comparison of mid‐ventricular short‐axis septal myocardium values for proposed $T_{1\rho }$ and 2D $T_{1\rho }$‐prep bSSFP across healthy subjects. Top/Bottom figures are the Mean/Standard Deviation (SD) of myocardial values. Left to Right are Bar chart, Violin, and Bland‐Altman plots.

**FIGURE 7:**
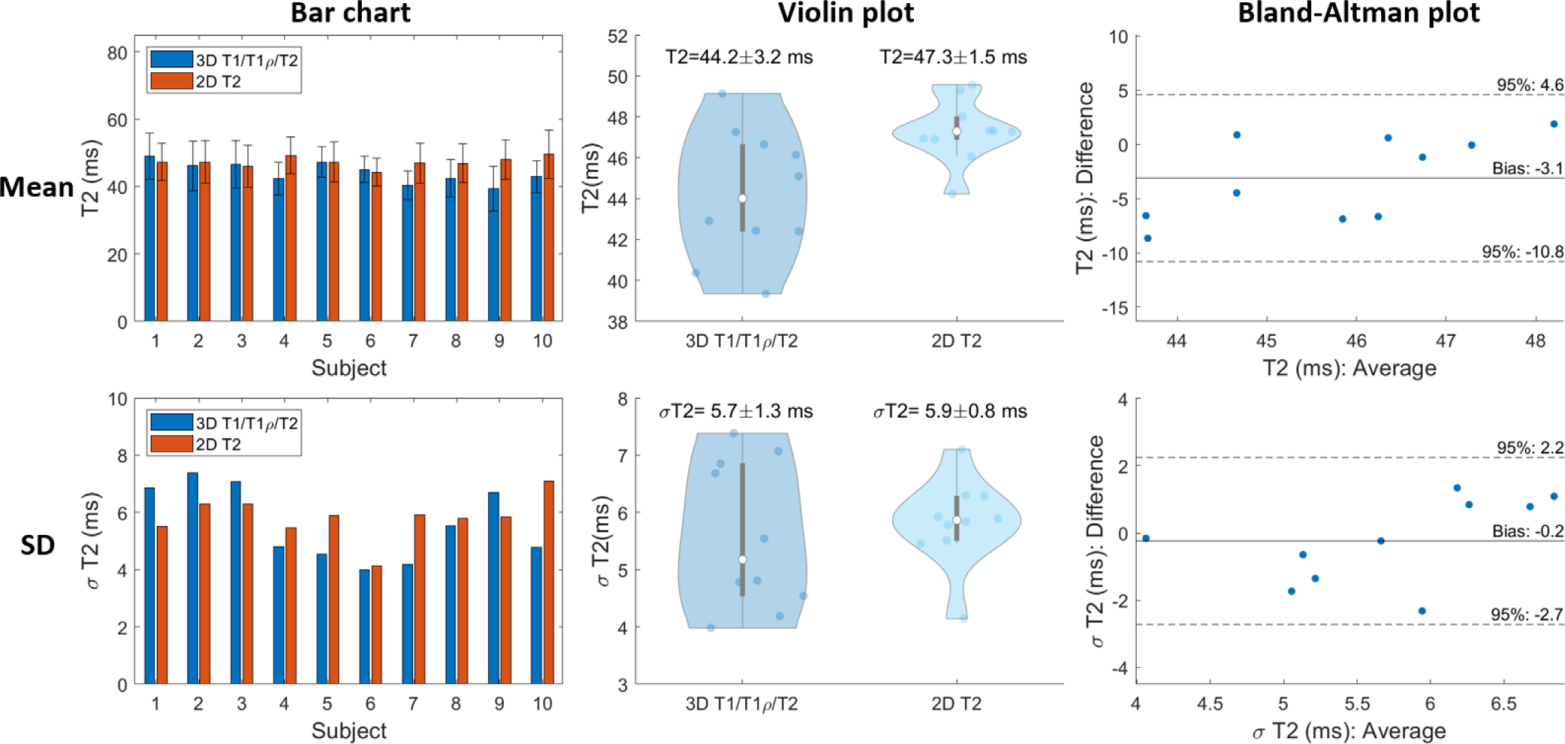
Comparison of mid‐ventricular short‐axis septal myocardium values for proposed $T_{2}$ and 2D $T_{2}$‐prep bSSFP across healthy subjects. Top/Bottom figures are the Mean/Standard Deviation (SD) of myocardial values. Left to Right are Bar chart, Violin, and Bland‐Altman plots.

For all subjects, the myocardium of a mid‐SAx slice was segmented for the proposed 3D $T_{1}$/$T_{1\rho }$/$T_{2}$ mapping, 2D MOLLI, 2D $T_{1\rho }$‐prep bSSFP, and 2D $T_{2}$‐prep bSSFP. Figures 5, 6, 7 illustrate a comparison of mid‐SAx septal myocardium values between the proposed and reference sequences across all healthy subjects for $T_{1}$, $T_{1\rho }$, and $T_{2}$, respectively. For $T_{1}$, mean septal values for the proposed 3D $T_{1}$/$T_{1\rho }$/$T_{2}$ sequence (1060±48ms) were significantly higher (p<0.0001) than for 2D MOLLI (959±15ms). For $T_{1}$, SD for the proposed sequence (60±13ms) was significantly higher (p=0.004) than 2D MOLLI (43±5ms). For $T_{1\rho }$, the mean septal value across subjects for the proposed 3D $T_{1}$/$T_{1\rho }$/$T_{2}$ sequence (48.1±3.9ms) was significantly smaller (p<0.0001) than 2D $T_{1\rho }$‐prep bSSFP (56.4±1.9ms). For $T_{1\rho }$, SD for the proposed sequence (5.2±0.9ms) was significantly smaller (p=0.002) than 2D $T_{1\rho }$‐prep bSSFP (6.7±0.9ms). For $T_{2}$, the mean septal value across subjects for the proposed 3D $T_{1}$/$T_{1\rho }$/$T_{2}$ sequence (44.2±3.2ms) was significantly smaller (p=0.02) than 2D $T_{2}$‐prep bSSFP (47.3±1.5ms). For $T_{2}$, SD for the proposed sequence (5.7±1.3ms) was slightly smaller than 2D $T_{2}$‐prep bSSFP (5.9±0.8ms), but not significantly.

**FIGURE 8:**
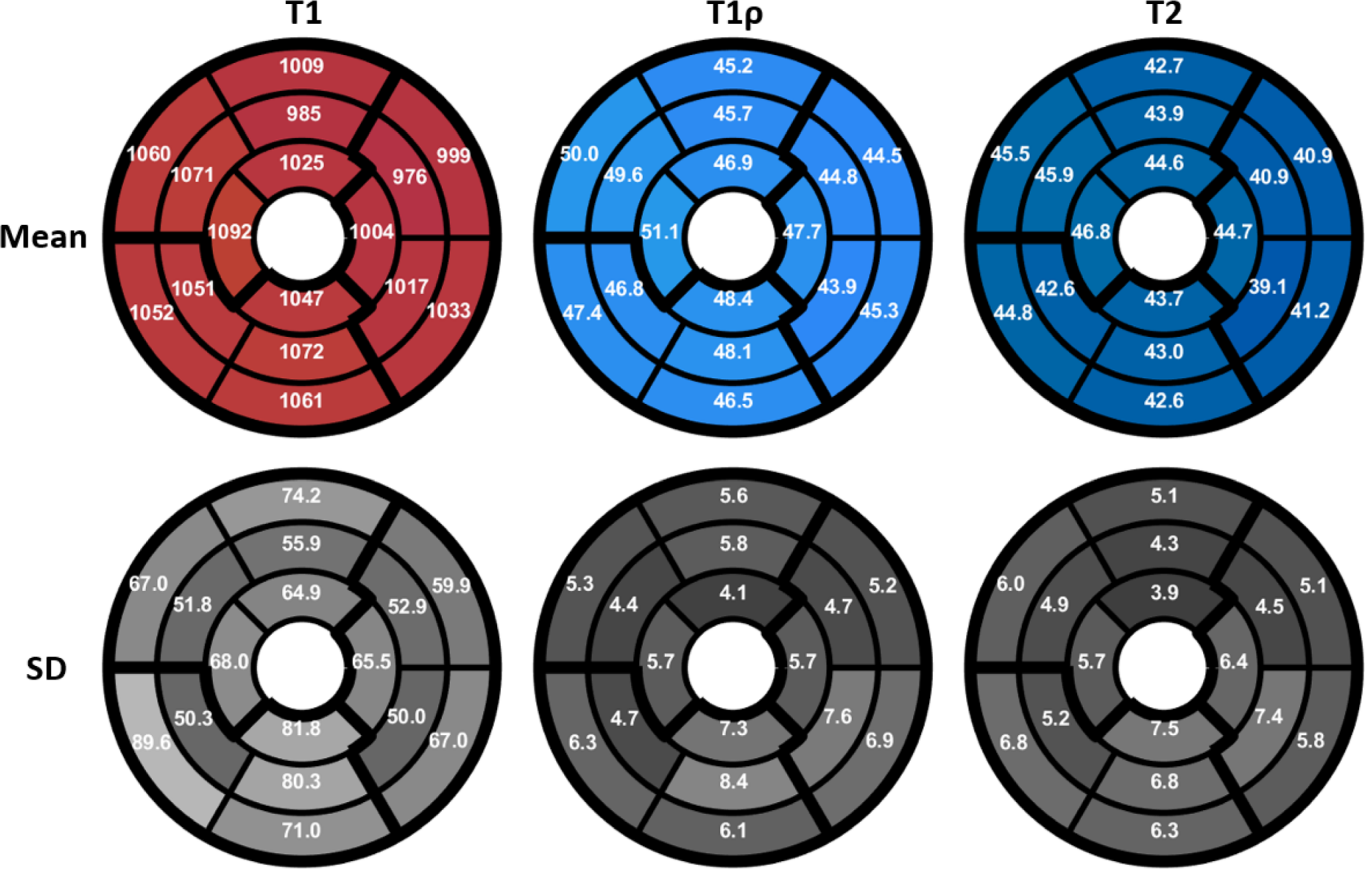
Bull's eye plots of Mean and Standard Deviation (SD) for $T_{1}$, $T_{1\rho }$, and $T_{2}$ (ms) for proposed 3D joint $T_{1}$/$T_{1\rho }$/$T_{2}$ mapping averaged across all 10 healthy subjects.

Bull's eye plots of mean/SD for $T_{1}$/$T_{1\rho }$/$T_{2}$ averaged across all healthy subjects are shown for proposed 3D joint $T_{1}$/$T_{1\rho }$ /$T_{2}$ mapping in Figure 8, demonstrating uniform $T_{1}$, $T_{1\rho }$, and $T_{2}$ values over the 16 AHA segments. Violin plots of mean and SD for $T_{1}$, $T_{1\rho }$, and $T_{2}$ for each AHA segment across all subjects are shown in Supporting Information Figure S7. Left‐ventricle myocardium values were computed for the proposed sequence, averaged across all healthy subjects. For $T_{1}$ Mean/SD = 1034±19ms/83.0±15.8ms, for $T_{1\rho }$ Mean/SD = 47.0±1.6ms/7.2±1.6ms, and for $T_{2}$ Mean/SD = 43.3±1.6ms/7.0±1.4ms. Mean/SD $T_{1}$, $T_{1\rho }$, and $T_{2}$ values across all healthy subjects for different regions of the heart are tabulated in Supporting Information Table S1.

We compare values between mid, base, and apex segments. Mean $T_{1}$ in base, mid, and apex segments was calculated to be 1036±24ms, 1029±24ms, and 1042±25ms. SD $T_{1}$ in base, mid, and apex segments was calculated to be 72±14ms, 58±13ms, and 70±16ms. There were no significant differences for mean or SD between these segments, apart from SD $T_{1}$ base which was significantly higher than mid (p=0.03). Mean $T_{1\rho }$ in base, mid, and apex segments was calculated to be 46.5±2.0ms, 46.5±2.3ms, and 48.5±1.8ms. SD $T_{1\rho }$ in base, mid and apex segments was calculated to be 5.9±1.2ms, 6.1±1.5ms, and 5.8±1.0ms. There were no significant differences for mean or SD between these segments, apart from mean $T_{1\rho }$ apex was significantly higher than mid (p=0.04) and base (p=0.03). Mean $T_{2}$ in base, mid and apex segments was calculated to be 42.9±1.5ms, 42.6±1.8ms, and 45.0±2.3ms. SD $T_{2}$ in base, mid and apex segments was calculated to be 5.9±1.3ms, 5.6±1.8ms, and 6.0±1.4ms. There was no significant differences for mean or SD between these segments, apart from mean $T_{2}$ for apex, which was significantly higher than mid (p=0.02) and base (p=0.03).

We now compare values between anterior, inferior, septal, and lateral segments. Mean $T_{1}$ in anterior, inferior, septal, and lateral segments was calculated to be 1007±23ms, 1060±39ms, 1065±37ms, and 1005±21ms. SD $T_{1}$ in anterior, inferior, septal, and lateral segments was calculated to be 65±14ms, 78±18ms, 67±11ms, and 60±16ms. Comparing septal and lateral segments, and anterior and inferior segments, mean $T_{1}$ septal was significantly larger (p<0.001) than lateral $T_{1}$, and mean $T_{1}$ inferior was significantly larger (p=0.002) than anterior $T_{1}$. Mean $T_{1\rho }$ in anterior, inferior, septal, and lateral segments was calculated to be 45.9±3.3ms, 47.7±3.6ms, 49.0±2.6ms, and 45.2±2.0ms. SD $T_{1\rho }$ in anterior, inferior, septal, and lateral segments was calculated to be 5.2±1.6ms, 7.3±2.0ms, 5.4±0.8ms, and 6.1±1.6ms. Comparing septal and lateral segments, and anterior and inferior segments, the mean $T_{1\rho }$ septal was significantly larger (p=0.002) than lateral $T_{1\rho }$, and SD $T_{1\rho }$ inferior was significantly larger (p=0.02) than anterior $T_{1\rho }$. Mean $T_{2}$ in anterior, inferior, septal, and lateral segments was calculated to be 43.8±3.0ms, 43.1±3.4ms, 45.1±2.2ms, and 41.4±1.4ms. SD $T_{2}$ in anterior, inferior, septal, and lateral segments was calculated to be 4.5±1.0ms, 6.9±2.3ms, 5.7±1.2ms, and 5.9±1.6ms. Comparing septal and lateral segments and anterior and inferior segments, mean $T_{2}$ septal was significantly larger (p<0.001) than lateral $T_{2}$, and SD $T_{2}$ inferior was significantly larger (p=0.02) than anterior $T_{2}$.

### 3.3 Patients

**FIGURE 9:**
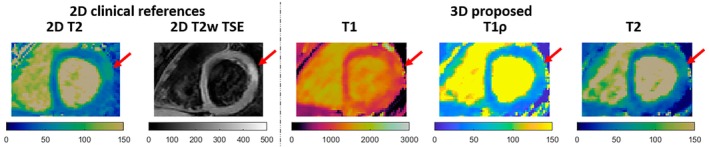
Comparison of 3D $T_{1}$/$T_{1\rho }$/$T_{2}$ with 2D LGE at a basal SAx slice for a patient with active myocarditis. Increased myocardial signal intensity can be observed in both the clinical 2D $T_{2}$ map and 2D $T_{2}$‐weighted Turbo Spin Echo ($T_{2}$w‐TSE) images. The proposed sequence indicates elevated $T_{2}$ (as well as $T_{1\rho }$ and $T_{1}$) in the corresponding region.

Results from a patient with active myocarditis involving the left ventricle lateral wall are shown in Figure 9. Increased myocardial signal intensity in the basal‐anterolateral segments is observed in the clinical reference 2D $T_{2}$ maps and 2D $T_{2}$w‐TSE images in the first 2 columns. The proposed 3D $T_{1}$/$T_{1\rho }$/$T_{2}$ mapping indicates elevated $T_{2}$ values (as well as $T_{1\rho }$ and $T_{1}$ values) in the corresponding region. Remote/basal‐anterolateral $T_{2}$ values were 45±2ms/78±4ms and 42±5ms/66±3ms for 2D reference and 3D proposed, respectively. Remote/basal‐anterolateral $T_{1}$ and $T_{1\rho }$ values for the proposed sequence were estimated to be: $T_{1}=1081\pm 55\text{ms}$/1319±69ms and $T_{1\rho }=46\pm 2\;\text{ms}$/71±3ms respectively.

## 4 DISCUSSION

A novel free‐breathing, 3D joint $T_{1}$/$T_{1\rho }$/$T_{2}$ mapping sequence with Dixon encoding has been proposed to provide co‐registered 3D $T_{1}$, $T_{1\rho }$, and $T_{2}$ maps and water‐fat volumes with isotropic spatial resolution in a single ≈7 min scan for comprehensive contrast‐agent‐free myocardial tissue characterization and simultaneous evaluation of the whole‐heart anatomy. The proposed sequence is an extension of the sequence proposed in^28^ but with additional $T_{1\rho }$ magnetization preparation, since $T_{1\rho }$ mapping has shown to be more sensitive to the detection of fibrotic tissue. The proposed sequence also uses a $2^{\text{nd}}$ angle increment across contrasts to increase the incoherence of undersampling artifacts in order to improve the performance of the patch‐based low‐rank regularization algorithm and enable higher undersampling factors. Additionally, the proposed framework has been extended to incorporate non‐rigid respiratory motion correction to the reconstructed volumes and subsequent maps.

The proposed free‐breathing mapping sequence provides 3D whole‐heart co‐registered water and fat volumes and $T_{1}$, $T_{1\rho }$, and $T_{2}$ maps in a clinically feasible time, addressing some of the previously mentioned limitations of 2D clinical mapping sequences acquired during a typical CMR examination. In phantoms, the proposed technique demonstrated good correlation with reference $T_{1}/T_{1\rho }/T_{2}$ SE values with a high coefficient of determination after a linear fit. The proposed sequence was compared to *in‐vivo* 2D MOLLI, 2D $T_{1\rho }$‐prep bSSFP, and 2D $T_{2}$‐prep bSSFP mapping sequences in 10 healthy subjects. For $T_{1}$, statistically significant larger mean and SD values were observed with proposed mapping than 2D MOLLI. The bias in mean $T_{1}$ could be explained by the known underestimation of $T_{1}$ by MOLLI.^56^ For $T_{1\rho }$ and $T_{2}$, statistically significant shorter mean values were observed with proposed mapping compared to reference 2D $T_{1\rho }$‐prep bSSFP and 2D $T_{2}$‐prep bSSFP, respectively, but the latter methods could be overestimating due to the unaccounted effect of imaging pulses during bSSFP readout.^57^ A whole‐heart analysis over all healthy subjects demonstrated some differences when comparing mapping values in basal, mid, and apical segments, with mean $T_{1\rho }$ and $T_{2}$ for apical segments being significantly shorter than mid and basal segments (p=0.02−0.04). Comparing anterior and inferior segments, mean $T_{1}$ inferior were significantly larger than anterior (p=0.002), and SD $T_{1\rho }$ and $T_{2}$ inferior was significantly larger than anterior (p=0.02). Comparing septal and lateral segments, mapping values in lateral segments were significantly shorter than septal segments for $T_{1}$ (p<0.001), $T_{1\rho }$ (p=0.002), and $T_{2}$ (p<0.001). Similar underestimation has been reported previously^22^, ^28^ and may be due to greater susceptibility artifacts at the lung‐myocardium interface and reduced coil sensitivity in lateral segments.^58^ The proposed sequence was acquired in 1 patient with active myocarditis and demonstrated good correlation with clinical 2D $T_{2}$‐prep bSSFP and 2D $T_{2}$w‐TSE.

This study has some limitations. First, the sequence could be sensitive to arrhythmias, since the assumed steady‐state signal would be affected by arrhythmic beats, potentially causing a loss of accuracy and precision in mapping values. The integration of prospective and retrospective arrhythmia rejection will be considered in future work. Second, only a small number of healthy subjects and patients were scanned, but further work will include acquisition of a larger cohort of healthy subjects and patients with different cardiovascular diseases to assess the suitability of the proposed mapping sequence for myocardial tissue characterization. Third, coils were manually selected in this work, but in future automatic coil selection techniques will be considered.^59^ Fourth, the sequence has potentially reduced $T_{2}$ and $T_{1\rho }$ sensitivity since there is only a single $T_{2}$‐prep and $T_{1\rho }$‐prep pulse per repetition, potentially limiting clinical usage of the sequence. Further work will include the use of an additional $T_{1\rho }$‐prep and $T_{2}$‐prep module to increase the encoding strength and improve sensitivity to $T_{1\rho }$ and $T_{2}$, at the expense of a longer 7 HB preparation sequence repetition. To offset the increase in scan time that would result, a further increase in VD‐CASPR acceleration or reduction of FOV (e.g., changing orientation to 3D short‐axis) will be explored. Additionally, an increase in spatial resolution would be considered to improve image quality. Super‐resolution techniques based on an end‐to‐end deep learning non‐rigid motion‐corrected reconstruction network will be considered to maintain a clinically feasible scan time.^60^, ^61^, ^62^, ^63^

Finally, an SLA =400 Hz was used in this study, but increasing SLA up to ≈1 kHz could better match physical processes of macromolecular‐water interactions in infarcted regions of the myocardium and thus increase the sensitivity of the proposed mapping to detect fibrosis. The maximal SLA is limited both by the maximum power of the RF amplifier as well as SAR constraints. Hence, it may be desirable to perform $T_{1\rho }$‐mapping at lower‐field, where SAR limitations ($\text{SAR}\propto |B_{0}{|}^{2}$) are decreased, and a future study will aim to acquire the proposed mapping sequence at lower $B_{0}$ field strengths.

## 5 CONCLUSIONS

A novel free‐breathing, 3D joint $T_{1}$/$T_{1\rho }$/$T_{2}$ mapping sequence with Dixon encoding was proposed to provide co‐registered 3D $T_{1}$, $T_{1\rho }$ and $T_{2}$ maps and water‐fat volumes with isotropic spatial resolution in a single ≈ 7 min scan for comprehensive contrast‐agent free myocardial tissue characterization and simultaneous evaluation of the whole‐heart anatomy. 3D joint $T_{1}$/$T_{1\rho }$/$T_{2}$ mapping and fat imaging results demonstrate good quantitative agreement when comparing to 2D $T_{1}$, $T_{1\rho }$, and $T_{2}$ references in phantoms and 10 healthy subjects. Data was acquired in 1 patient with active myocarditis, and promising results were obtained when comparing it to conventional 2D $T_{2}$w‐TSE.

## CONFLICT OF INTEREST STATEMENT

K. P. Kunze is an employee of Siemens Healthcare Limited.

## Supporting information




**Data S1.** Supporting Information.
**Figure S1.** Simulation of sequence precision and accuracy, with *N*_*n*_ = 1000 noise realisations with SNR = 70 dB. Left to Right figures indicates (Bias) and Coefficient of Variation (CoV) for fixed *T*_1_ = 1100 ms, fixed *T*_1ρ_ = 56 ms, and fixed *T*_2_ = 44 ms. Top to Bottom are *T*_1_/*T*_1ρ_/*T*_2_ values.
**Figure S2.** Comparison of proposed 3D *T*_1_/*T*_1ρ_/*T*_2_ values to 2D *T*_1_ SE, 2D *T*_1ρ_ SE, and 2D *T*_2_ SE in *T*_1_‐MES and in‐house phantoms as function of simulated HR = [60, 80, 100, 120] bpm. Vials with *T*_1_ ≥ 1500 ms, *T*_1ρ_ ≥ 150 ms, *T*_2_ ≥ 150 ms were rejected to focus on typical myocardial tissue values. Left: Scatter plots including the coefficient of determination from a linear fit, and the black dashed lines are the identity line. Right: Bar charts of Mean and Coefficient of Variation (CoV) estimation for each vial for proposed 3D *T*_1_/*T*_1ρ_/*T*_2_ mapping and 2D SE references. Good agreement is observed between 3D proposed and 2D SE references with an *r* ^2^ > 0.993 over all simulated HR and *T*_1_/*T*_1ρ_/*T*_2_.
**Figure S3.** 3D Water and Fat images for each volume from the proposed 3D joint *T*_1_/*T*_1ρ_/*T*_2_ mapping sequence with Dixon encoding in mid‐coronal view for one representative healthy subject.
**Figure S4.** Whole‐heart analysis of *T*_1_ for one representative healthy subject (Subject 7): (A) *T*_1_ maps in short‐axis view from apex to base, (B) high‐resolution Bull's eye plots of Mean (normalised to mean across whole left ventricle myocardium)/Coefficient of Variation (CoV) *T*_1_ with the Mean(normalised)/CoV(%) of each AHA segment displayed, (C) histogram of *T*_1_ values across the whole left‐ventricle with measured *T*_1_ = 1039 ± 81 ms, CoV = 7.8%.
**Figure S5.** Whole‐heart analysis of *T*_1ρ_ for one representative healthy subject (Subject 7): (A) *T*_1ρ_ maps in short‐axis view from apex to base, (B) high‐resolution Bull's eye plots of Mean (normalised to mean across whole left ventricle myocardium)/Coefficient of Variation (CoV) *T*_1ρ_ with the Mean(normalised)/CoV(%) of each AHA segment displayed, (C) histogram of *T*_1ρ_ values across the whole left‐ventricle with measured *T*_1ρ_ = 46.7 ± 6.7 ms, CoV = 14.4%.
**Figure S6.** Whole‐heart analysis of *T*_2_ for one representative healthy subject (Subject 7): (A) *T*_2_ maps in short‐axis view from apex to base, (B) high‐resolution Bull's eye plots of Mean (normalised to mean across whole left ventricle myocardium)/Coefficient of Variation (CoV) *T*_2_ with the Mean(normalised)/CoV(%) of each AHA segment displayed, (C) histogram of *T*_2_ values across the whole left‐ventricle with measured *T*_2_ = 42.5 ± 6.5 ms, CoV = 15.3%.
**Figure S7.** Violin plots of Mean and Standard Deviation myocardium values for *T*_1_/*T*_1ρ_/*T*_2_ across all healthy subjects over all 16 AHA segments. Ba = Basal, Mi = Mid, Ap = Apex, A = Anterior, I = Inferior, S = Septal, L = Lateral.
**Table S1.** Mean/Standard Deviation (SD) myocardium *T*_1_, *T*_1ρ_ and *T*_2_ values of 10 healthy volunteers in different regions of the heart measured with the proposed 3D whole‐heart joint *T*_1_/*T*_1ρ_/*T*_2_ mapping sequence.
