# Supplementary material for: 3D joint T1/T1 ρ/T2 mapping and water‐fat imaging for contrast‐agent free myocardial tissue characterization at 1.5T
Source: Magn Reson Med. 2025 Feb 21;93(6):2297–310. doi: 10.1002/mrm.30397 (PMC11971512; doi:10.1002/mrm.30397)
Supplement: Supplementary file 1 — Data S1. Supporting Information. Figure S1. Simulation of sequence precision and accuracy, with N n = 1000 noise realisations with SNR = 70 dB. Left to Right figures indicates (Bias) and Coefficient of Variation (CoV) for fixed T 1 = 1100 ms, fixed T 1ρ = 56 ms, and fixed T 2 = 44 ms. Top to Bottom are T 1/T 1ρ/T 2 values. Figure S2. Comparison of proposed 3D T 1/T 1ρ/T 2 values to 2D T 1 SE, 2D T 1ρ SE, and 2D T 2 SE in T 1‐MES and in‐house phantoms as function of simulated HR = [60, 80, 100, 120] bpm. Vials with T 1 ≥ 1500 ms, T 1ρ ≥ 150 ms, T 2 ≥ 150 ms were rejected to focus on typical myocardial tissue values. Left: Scatter plots including the coefficient of determination from a linear fit, and the black dashed lines are the identity line. Right: Bar charts of Mean and Coefficient of Variation (CoV) estimation for each vial for proposed 3D T 1/T 1ρ/T 2 mapping and 2D SE references. Good agreement is observed between 3D proposed and 2D SE references with an r 2 > 0.993 over all simulated HR and T 1/T 1ρ/T 2. Figure S3. 3D Water and Fat images for each volume from the proposed 3D joint T 1/T 1ρ/T 2 mapping sequence with Dixon encoding in mid‐coronal view for one representative healthy subject. Figure S4. Whole‐heart analysis of T 1 for one representative healthy subject (Subject 7): (A) T 1 maps in short‐axis view from apex to base, (B) high‐resolution Bull's eye plots of Mean (normalised to mean across whole left ventricle myocardium)/Coefficient of Variation (CoV) T 1 with the Mean(normalised)/CoV(%) of each AHA segment displayed, (C) histogram of T 1 values across the whole left‐ventricle with measured T 1 = 1039 ± 81 ms, CoV = 7.8%. Figure S5. Whole‐heart analysis of T 1ρ for one representative healthy subject (Subject 7): (A) T 1ρ maps in short‐axis view from apex to base, (B) high‐resolution Bull's eye plots of Mean (normalised to mean across whole left ventricle myocardium)/Coefficient of Variation (CoV) T 1ρ with the Mean(normalised)/CoV(%) of each AHA se [file MRM-93-2297-s001.pdf]

S1: Sequence simulation

To test the sequence accuracy and precision, for any parameter combination $T_1/T_{1\rho}/T_2$, the sequence was simulated to form a signal evolution $s \in \mathbb{R}^5$. Additive white Gaussian noise with constant variance/contrast was included over N_n pseudo-random noise realisations to form a set of measurements $S \in \mathbb{R}^{5\times N_n}$. These measurements were then matched to the dictionary to generate N_n estimated parameters $\{(T_1, T_{1\rho}, T_2)\}_i \; i = 1, \ldots, N_n$. The Bias/Coefficient of Variation (CoV) were estimated for each parameters through the following

$$\text{Bias}(Y) = \mu_Y - Y, \qquad\qquad \text{CoV}(Y) = \sigma_Y/\mu_Y, \tag{1}$$

for $Y = T_1/T_{1\rho}/T_2$, and μ_Y, σ_Y are the mean and standard-deviation of Y respectively. This process was repeated for each parameter combination of interest $\{(T_1, T_{1\rho}, T_2)\}_p$, $p = 1, \ldots, N_p$. For the simulation, $N_n = 1000$ noise additions were included with SNR = 70 dB, and the parameter space of interest selected as $T_1 = [900 : 50 : 1400]$ ms, $T_{1\rho} = [40 : 4 : 80]$ ms, $T_2 = [32 : 4 : 60]$ ms matching typical myocardial tissue values at 1.5T. A simulated HR= 60 bpm and acquisition window = 120 ms were used. Fig. S1 indicates a plot of |Bias| and CoV of the 3D parameter space for fixed $T_1 = 1100$ ms, fixed $T_{1\rho} = 56$ ms, and fixed $T_2 = 44$ ms, which demonstrates generally low bias and low CoV for T_1, $T_{1\rho}$ and T_2 over the parameter range simulated.

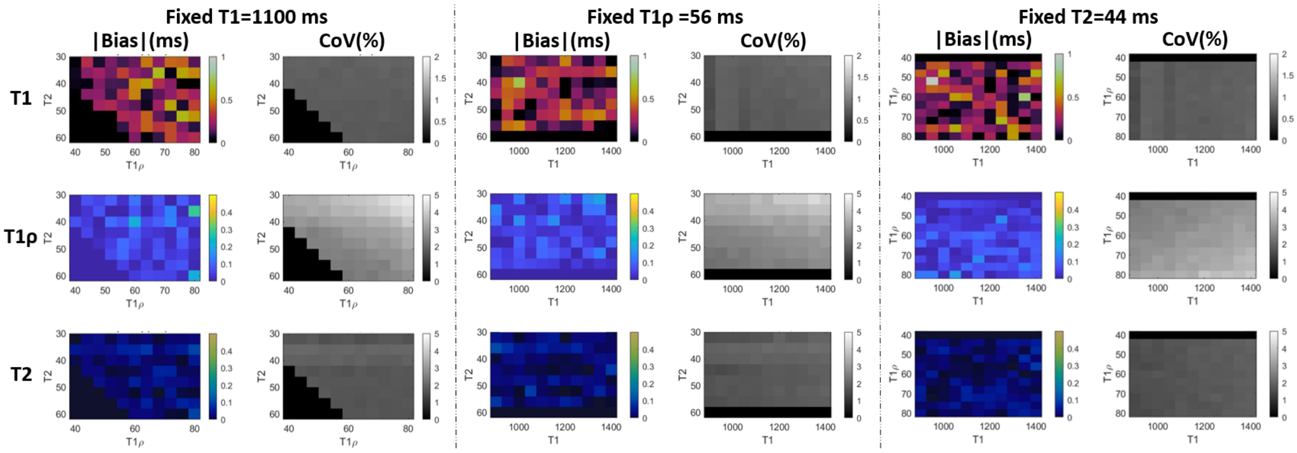

Figure S1: Simulation of sequence precision and accuracy, with $N_n = 1000$ noise realisations with SNR=70 dB. Left to Right figures indicates |Bias| and Coefficient of Variation (CoV) for fixed $T_1 = 1100$ ms, fixed $T_{1\rho} = 56$ ms, and fixed $T_2 = 44$ ms. Top to Bottom are $T_1/T_{1\rho}/T_2$ values.

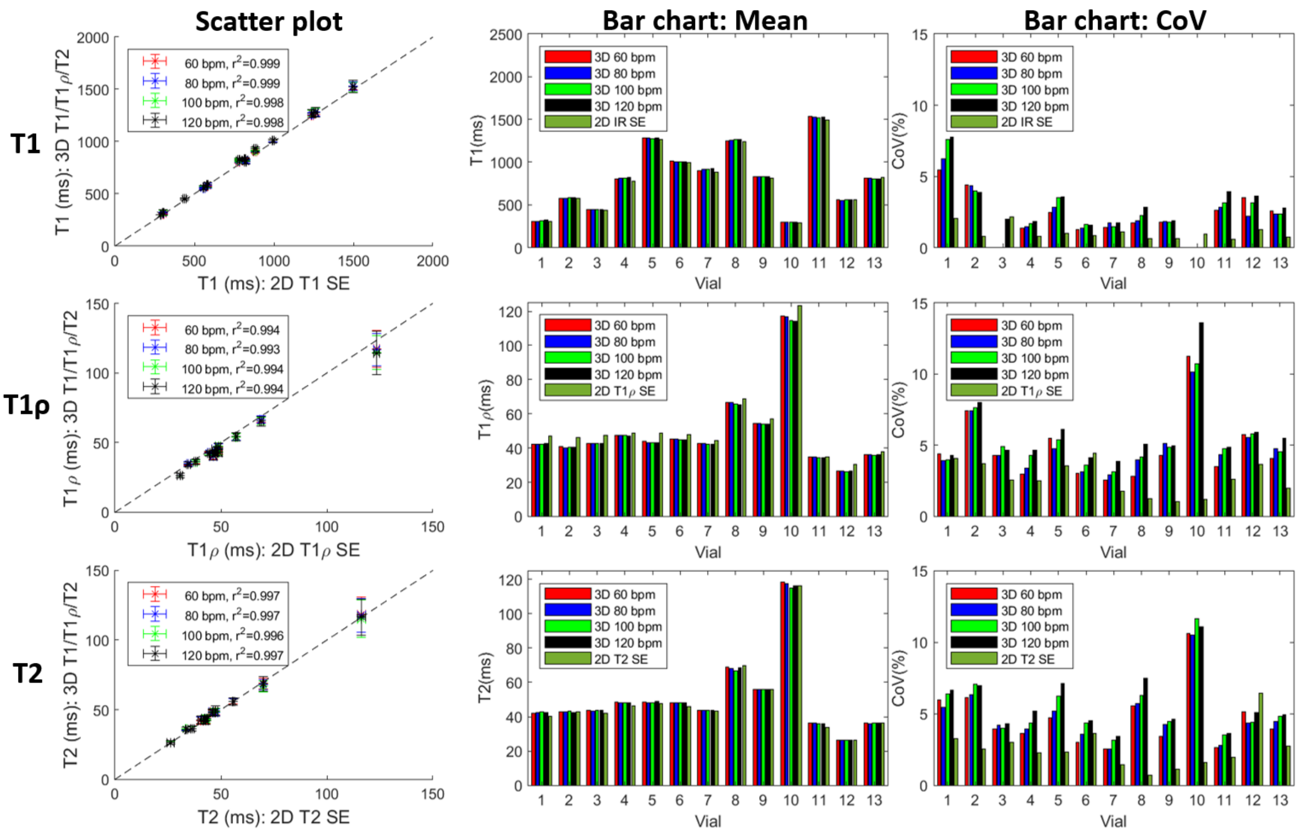

Figure S2: Comparison of proposed 3D $T_1/T_{1\rho}/T_2$ values to 2D T_1 SE, 2D $T_{1\rho}$ SE, and 2D T_2 SE in T_1-MES and in-house phantoms as function of simulated HR$= [60, 80, 100, 120]$ bpm. Vials with $T_1 \geq 1500$ ms, $T_{1\rho} \geq 150$ ms, $T_2 \geq 150$ ms were rejected to focus on typical myocardial tissue values. Left: Scatter plots including the coefficient of determination from a linear fit, and the black dashed lines are the identity line. Right: Bar charts of Mean and Coefficient of Variation (CoV) estimation for each vial for proposed 3D $T_1/T_{1\rho}/T_2$ mapping and 2D SE references. Good agreement is observed between 3D proposed and 2D SE references with an $r^2 > 0.993$ over all simulated HR and $T_1/T_{1\rho}/T_2$.

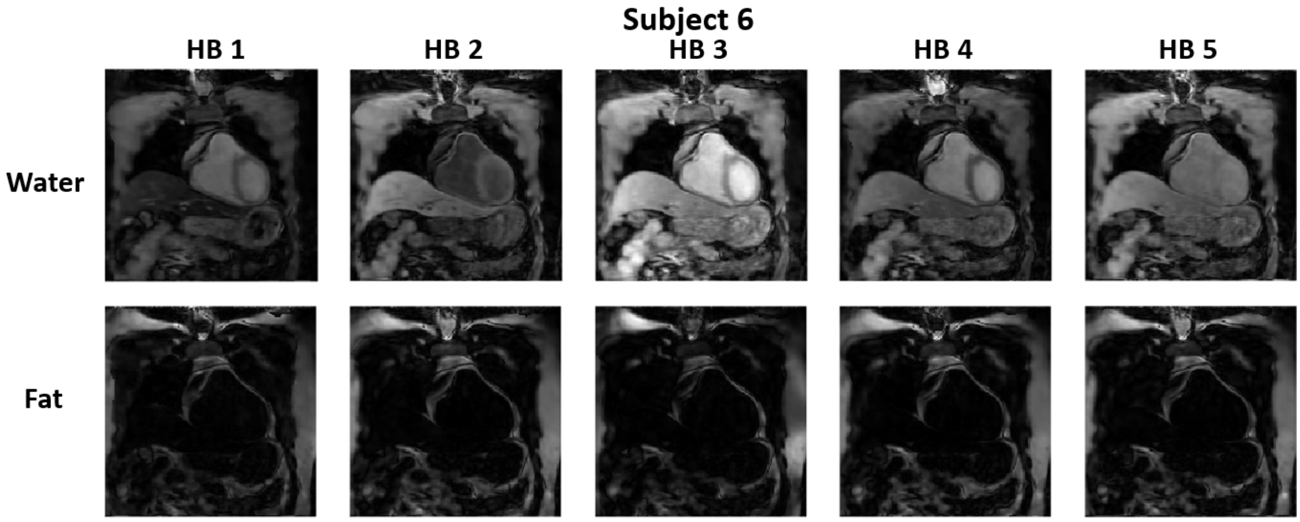

Figure S3: 3D Water and Fat images for each volume from the proposed 3D joint $T_1/T_{1\rho}/T_2$ mapping sequence with Dixon encoding in mid-coronal view for one representative healthy subject.

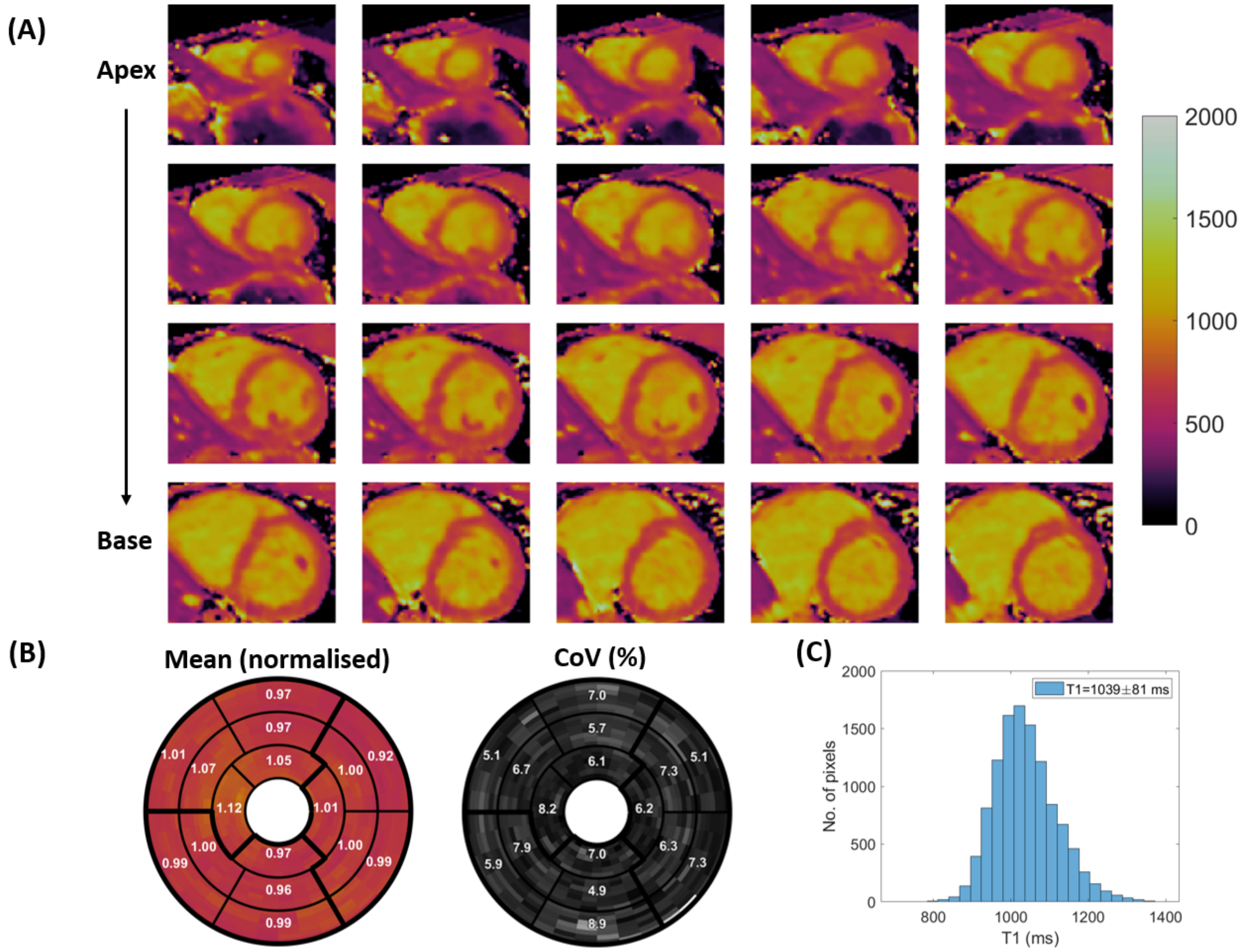

Figure S4: Whole-heart analysis of T_1 for one representative healthy subject (Subject 7): (A) T_1 maps in short-axis view from apex to base, (B) high-resolution Bull's eye plots of Mean (normalised to mean across whole left ventricle myocardium)/Coefficient of Variation (CoV) T_1 with the Mean(normalised)/CoV(%) of each AHA segment displayed, (C) histogram of T_1 values across the whole left-ventricle with measured $T_1 = 1039 \pm 81$ ms, CoV=7.8%.

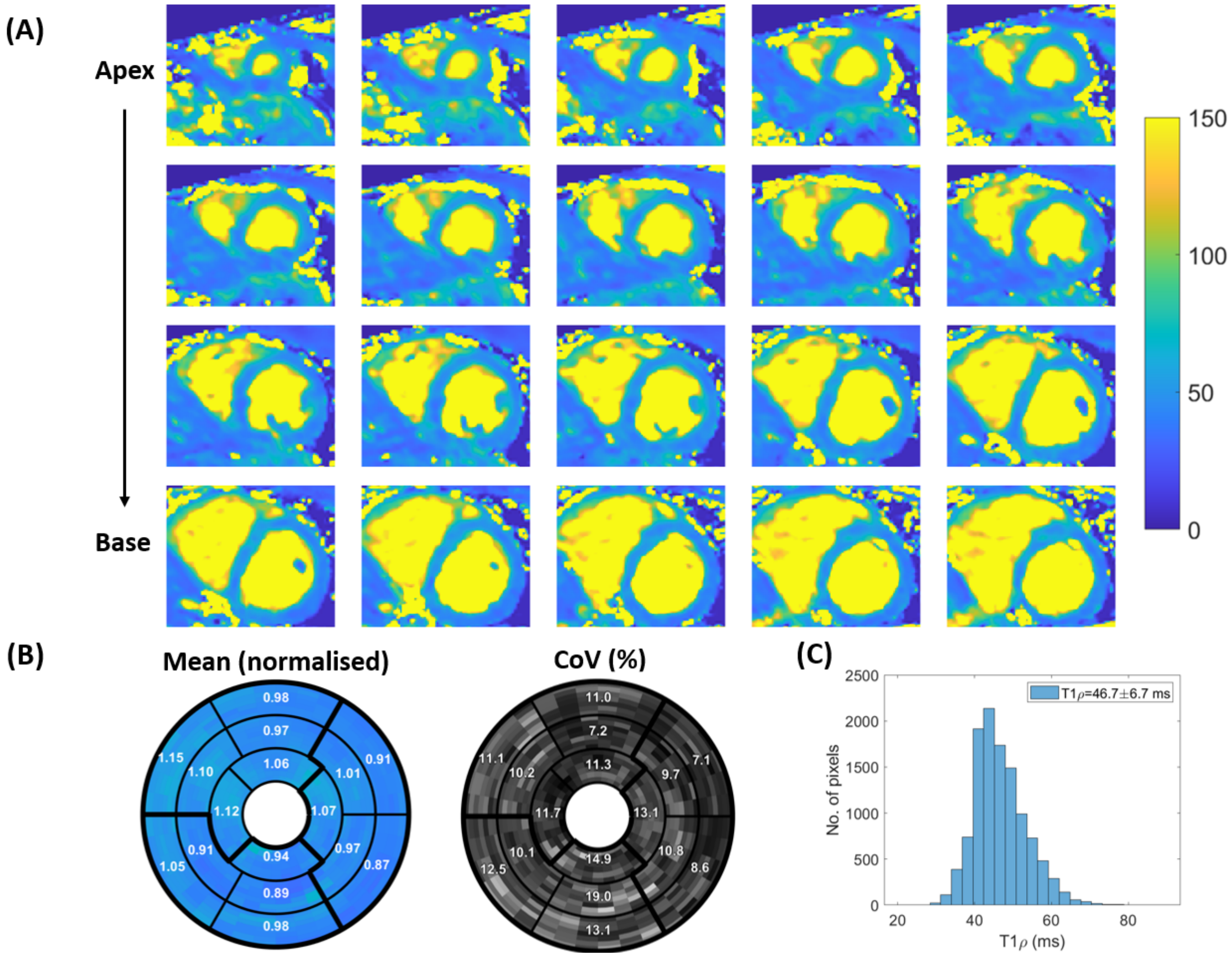

Figure S5: Whole-heart analysis of $T_{1\rho}$ for one representative healthy subject (Subject 7): (A) $T_{1\rho}$ maps in short-axis view from apex to base, (B) high-resolution Bull's eye plots of Mean (normalised to mean across whole left ventricle myocardium)/Coefficient of Variation (CoV) $T_{1\rho}$ with the Mean(normalised)/CoV(%) of each AHA segment displayed, (C) histogram of $T_{1\rho}$ values across the whole left-ventricle with measured $T_{1\rho} = 46.7 \pm 6.7$ ms, CoV=14.4%.

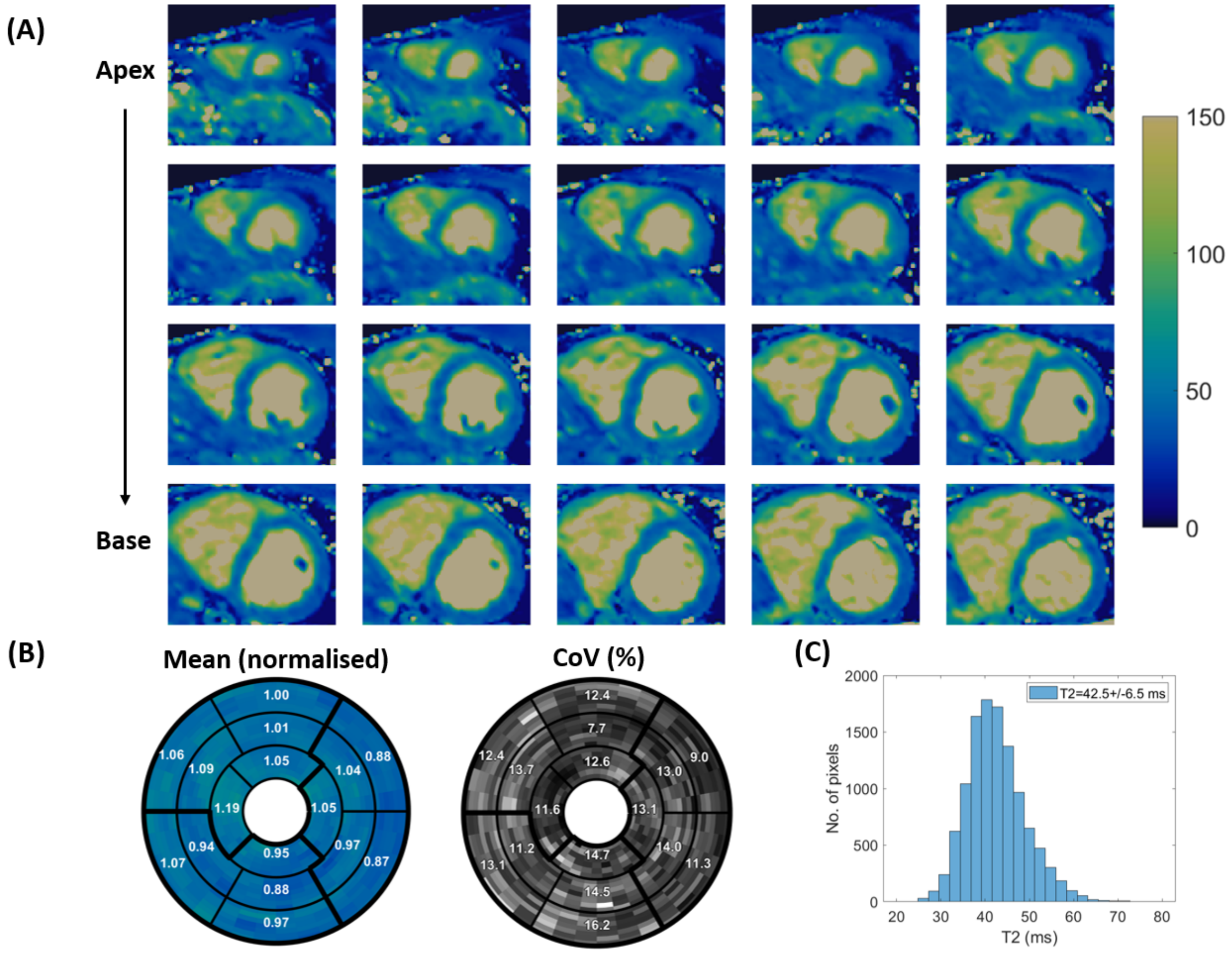

Figure S6: Whole-heart analysis of T_2 for one representative healthy subject (Subject 7): (A) T_2 maps in short-axis view from apex to base, (B) high-resolution Bull's eye plots of Mean (normalised to mean across whole left ventricle myocardium)/Coefficient of Variation (CoV) T_2 with the Mean(normalised)/CoV(%) of each AHA segment displayed, (C) histogram of T_2 values across the whole left-ventricle with measured $T_2 = 42.5 \pm 6.5$ ms, CoV=15.3%.

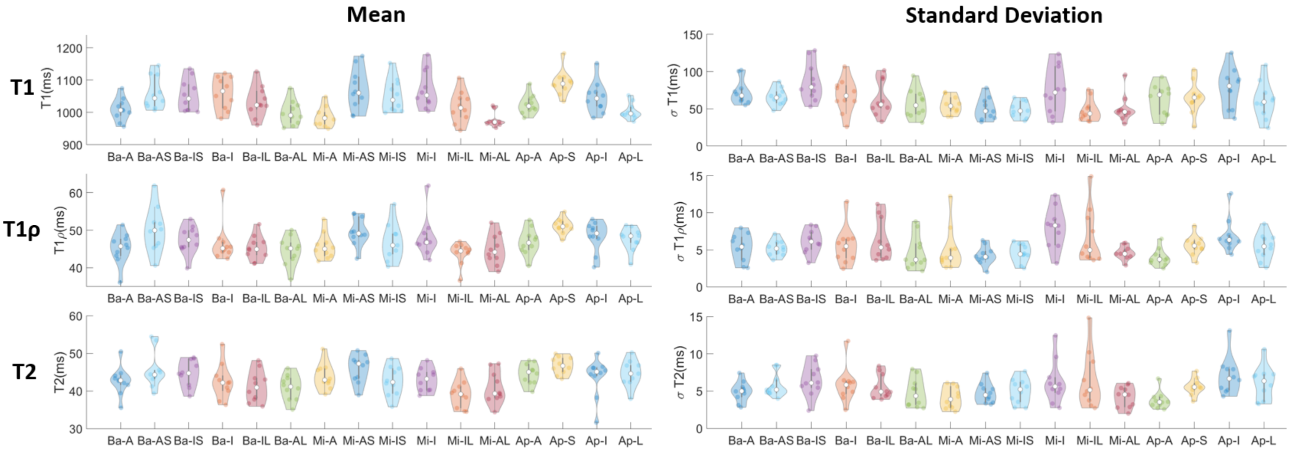

Figure S7: Violin plots of Mean and Standard Deviation myocardium values for $T_1/T_{1\rho}/T_2$ across all healthy subjects over all 16 AHA segments. Ba=Basal, Mi=Mid, Ap=Apex, A=Anterior, I=Inferior, S=Septal, L=Lateral.

	**Mean**			**SD**		
	$\mathbf{T_1}$**(ms)**	$\mathbf{T_{1\rho}}$**(ms)**	$\mathbf{T_2}$**(ms)**	$\mathbf{T_1}$**(ms)**	$\mathbf{T_{1\rho}}$**(ms)**	$\mathbf{T_2}$**(ms)**
**Mid-Septal**	1060 ± 48	48.1 ± 3.9	44.2 ± 3.2	60.0 ± 12.9	5.2 ± 0.9	5.7 ± 1.3
**Apex**	1042 ± 25	48.5 ± 1.8	45.0 ± 2.3	70.4 ± 16.4	5.8 ± 1.0	6.0 ± 1.4
**Mid**	1029 ± 24	46.5 ± 2.3	42.6 ± 1.8	57.9 ± 13.4	6.1 ± 1.5	5.6 ± 1.8
**Base**	1036 ± 24	46.5 ± 2.0	42.9 ± 1.5	72.0 ± 13.8	5.9 ± 1.2	5.9 ± 1.3
**Anterior**	1007 ± 23	45.9 ± 3.3	43.8 ± 3.0	65.4 ± 14.4	5.2 ± 1.6	4.5 ± 1.0
**Inferior**	1060 ± 39	47.7 ± 3.6	43.1 ± 3.4	77.8 ± 17.5	7.3 ± 2.0	6.9 ± 2.3
**Septal**	1065 ± 37	49.0 ± 2.6	45.1 ± 2.2	66.9 ± 10.8	5.4 ± 0.8	5.7 ± 1.2
**Lateral**	1006 ± 21	45.2 ± 2.0	41.4 ± 1.4	59.5 ± 15.5	6.1 ± 1.6	5.9 ± 1.6
**Left-ventricle**	1034 ± 19	47.0 ± 1.6	43.3 ± 1.6	83.0 ± 15.8	7.2 ± 1.6	7.0 ± 1.4

Table S1: Mean/Standard Deviation (SD) myocardium T_1, $T_{1\rho}$ and T_2 values of 10 healthy volunteers in different regions of the heart measured with the proposed 3D whole-heart joint $T_1/T_{1\rho}/T_2$ mapping sequence.
